# Key aspects for conception and construction of co-culture models of tumor-stroma interactions

**DOI:** 10.3389/fbioe.2023.1150764

**Published:** 2023-04-07

**Authors:** James Mason, Daniel Öhlund

**Affiliations:** ^1^ Department of Radiation Sciences, Umeå University, Umeå, Sweden; ^2^ Wallenberg Centre for Molecular Medicine, Umeå University, Umeå, Sweden

**Keywords:** co-culture, organoid, cell culture models, cancer associated fibroblasts, tumor-stroma interactions

## Abstract

The tumor microenvironment is crucial in the initiation and progression of cancers. The interplay between cancer cells and the surrounding stroma shapes the tumor biology and dictates the response to cancer therapies. Consequently, a better understanding of the interactions between cancer cells and different components of the tumor microenvironment will drive progress in developing novel, effective, treatment strategies. Co-cultures can be used to study various aspects of these interactions in detail. This includes studies of paracrine relationships between cancer cells and stromal cells such as fibroblasts, endothelial cells, and immune cells, as well as the influence of physical and mechanical interactions with the extracellular matrix of the tumor microenvironment. The development of novel co-culture models to study the tumor microenvironment has progressed rapidly over recent years. Many of these models have already been shown to be powerful tools for further understanding of the pathophysiological role of the stroma and provide mechanistic insights into tumor-stromal interactions. Here we give a structured overview of different co-culture models that have been established to study tumor-stromal interactions and what we have learnt from these models. We also introduce a set of guidelines for generating and reporting co-culture experiments to facilitate experimental robustness and reproducibility.

## 1 Introduction

Cancer remains a major cause of mortality worldwide and is the second leading cause of death in the United States and Europe, where it is responsible for approximately 2 million deaths combined annually (Malvezzi et al., 2019; Siegel et al., 2023). Understanding of the diseases collectively termed cancer has vastly improved. It has become clear that tumors are not simply a direct function of the mutation-carrying cancer cells themselves, but that their development is shaped by iterative interaction with their surrounding microenvironment. The tumor microenvironment (TME) is constructed both by the cancer cells and the surrounding stroma. It is composed of many factors instructive to tumor behaviour ranging from mechanical and physicochemical properties of the extracellular matrix (ECM) to signaling between neighboring cells (Balkwill et al., 2012).

**FIGURE 1:**
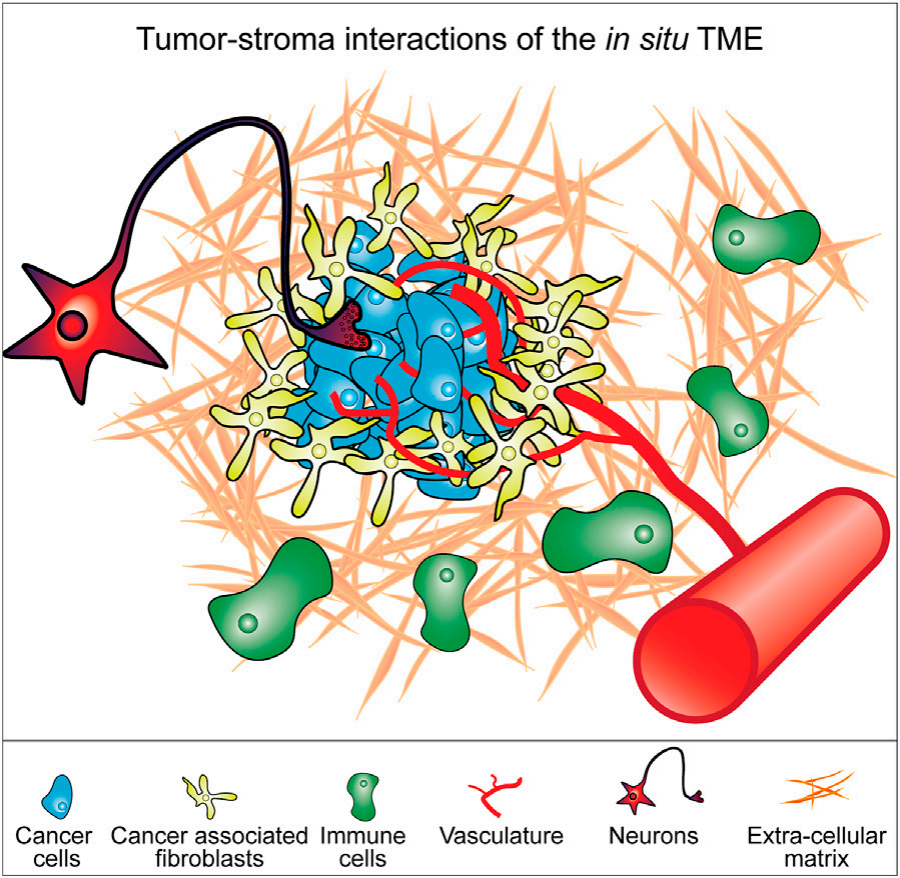
Model of constituent components of the tumor microenvironment (TME). Cancer cells exist in a microenvironment containing multiple different cell types and a particular extracellular matrix context that together form the tumor. The interplay between all these components determines the biology of the tumor. These various components represent elements that can be incorporated into cell culture models of cancer cells to better replicate the *in situ* tumor microenvironment.

In addition to the ECM, the TME contains a diverse milieu of many different cell types, including cancer associated fibroblasts (CAFs), immune cells, endothelial cells, neurons and others that intercommunicate with the cancer cells and each other, shaping the tumor niche (Figure 1) (Balkwill et al., 2012). The TME is therapeutically consequential, providing protections to the tumor such as immune cell exclusion as well as providing a variety of pro-proliferative signals (Balkwill et al., 2012; Arneth, 2019). During later stages of disease progression, the TME influences the metastatic potential of tumors, and could provide clues as to how to mitigate cancer cell migration and invasion as well as approaches to treat tumors at secondary sites (Subarsky and Hill, 2003; Joyce and Pollard, 2009; Aiello et al., 2016). Not all functions of the tumor-stroma are tumor-promoting and there is increasing recognition that some cell types in the TME act to restrain tumor growth. It has also been demonstrated that unspecific ablation of TME cell types such as CAFs can accelerate tumor progression and lead to worse patient outcomes in clinical trials (Madden, 2012; Rhim et al., 2014; Özdemir et al., 2014; Bausch et al., 2020).

It is clear, then, that understanding tumor biology, especially with respect to therapeutic application, requires an understanding of the TME which is so instructive to tumor behaviour. Potentially, these signals can be utilized to manipulate the TME to develop powerful anticancer therapeutics.

The three main tools used for exploring tumor biology are patient biopsies, animal models and *in vitro* cell culture models. These approaches are not mutually exclusive and can also be used in combination such as in xenograft models using transplantation of either derived cell lines or even patient tissue into recipient, typically murine, organisms. Indeed, the use of patient-derived orthotopic xenograft models has expanded to provide insight in both personalized and precision translational medicine [reviewed in (Hidalgo et al., 2014; Abdolahi et al., 2022)]. However, of these main tools, the most scalable and controllable for robust investigation are cell culture models. We have learned much from traditional two-dimensional (2D) monocultures of tumor-derived cell lines where cells are cultured in monolayers on a plastic surface. However, this approach has some severe limitations; not least the disheartening statistic that the vast majority of therapeutics developed against 2D tumor cell cultures fail to translate to clinic or even animal models (Scannell et al., 2012; Seyhan, 2019). In part, this is due to the insufficiency of 2D monoculture to appropriately model the TME.

**FIGURE 2:**
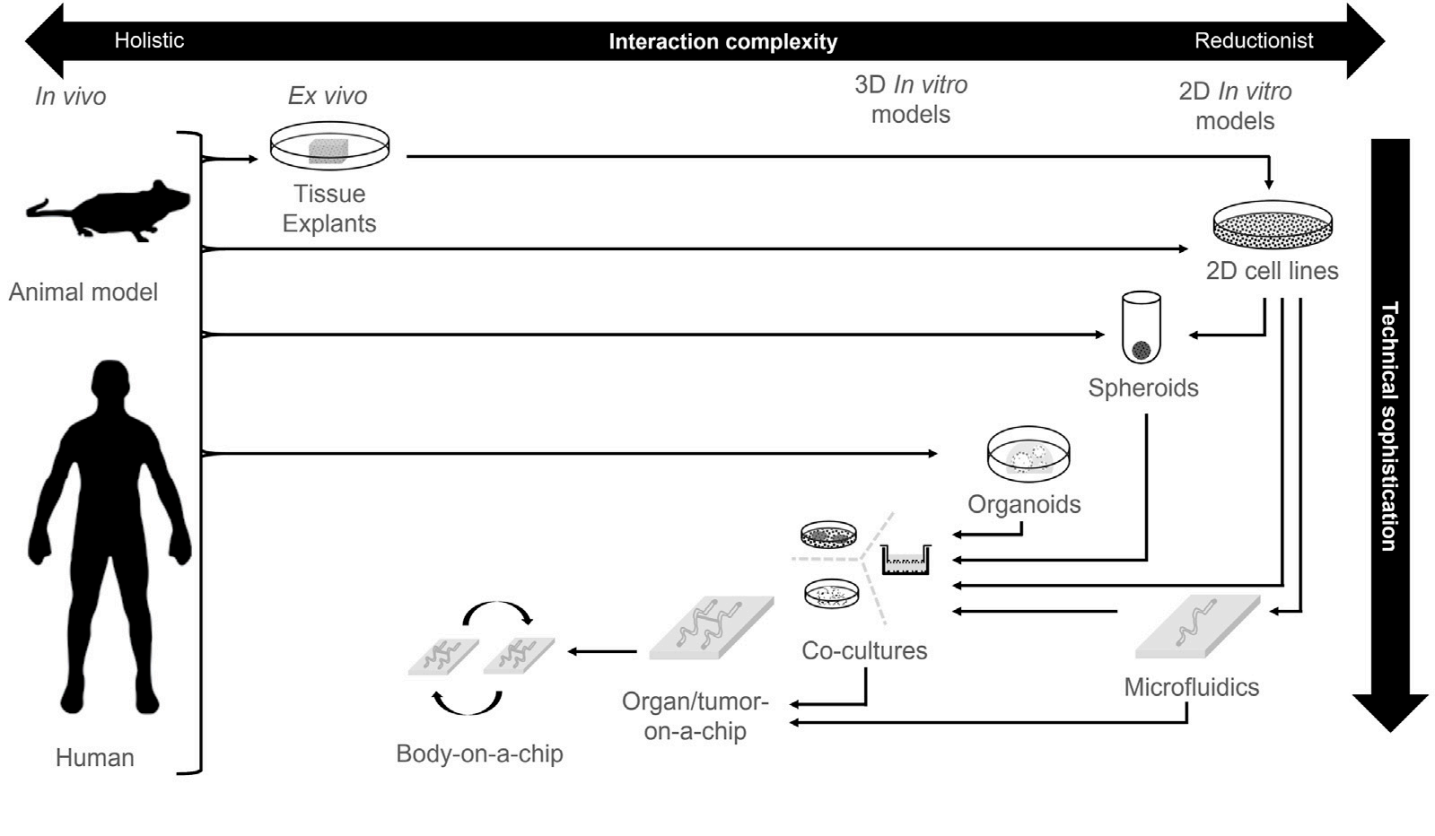
Technological sophistication of cell culture platforms. An overview of different cell culture technologies and the development of technical sophistication compared to biological complexity. Arrows represent typical model derivation routes.

Development of more representative cell culture models of both healthy and tumor contexts is an ongoing task of global ingenuity and technical accomplishment. Many of the major developments that make these models more resemble biological systems are discussed in this review (Figure 2; Table 1). For instance, the development of three-dimensional (3D) cell culture directly alters the mechanical signaling inputs for cells, whereas in 2D cultures on plastic these cells respond to stiff culture plastic surfaces. This innovation resulted in the generation of spheroid cultures whereby established cell lines can be cultivated in low-adherence settings that lead to self-aggregated multicellular spheres. The addition of ECM scaffolds of appropriate protein composition also provides instructive signaling cues as well as adjusted mechanical properties.

**TABLE 1:** Model types from Figure 2; sources, pros and cons. A summary of the models depicted in Figure 2, with an overview of their derivation, as well as experimental pros and cons. TME; Tumor microenvironment.

Model type	Source	Pros	Cons
**Human**	Patient/volunteer	High physiological relevance	Limited assays; expensive; Lacks robust control and replication
**Animal model**	Animal model	High physiological relevance; established (genetic) models improve reproducibility	Limited assays; expensive; established models highly inbred
**Tissue explants**	Biopsied tissue (human or animal model)	Complex; faithful recapitulation of *in situ* TME	Difficult long-term maintenance
**2D cell lines**	Biopsied tissue outgrowth/selection	Very scalable; many available assays	Significant loss of physiological (cellular and TME) complexity; Mechanical signaling context of culture plastic; Long term culture adaptation
**Spheroids**	2D cell lines cultured in spheroid conditions; direct establishment from biopsied tissue	Quite scalable; 3D arrangement and structure; compatible with appropriate ECM scaffolding; experimentally versatile	Low complexity; does not self-organize; singular cell type; poor TME recapitulation
**Organoids**	Establishment from biopsied tissue	Similarities to original organ; self-organizing 3D arrangement containing multiple cell types with polarity from stem cell population; experimentally versatile	Scalability varies between organ and species types; Specific culture/media conditions required; Expensive ECM scaffolding for maintenance
**Co-cultures**	Established cell/spheroid/organoid lines	Increased complexity resembling interactions seen physiologically in TME; Experimentally versatile; many available models	Characterization of cell types is more complicated; Readout interpretation confounded by presence of multiple cell types; Increased experimental variability
**Microfluidics**	Established cell/spheroid/organoid lines	More relevant model of media exchange resembling physiological TME setting; experimentally versatile	Technical model establishment with specialist equipment and chip design; Small volumes for subsequent analyses
**Organ/tumor-on-a-chip**	Co-culture arrangement(s) coupled with microfluidic media exchange	Relevant models of organ/tumor function including TME recapitulation	Technically challenging arrangement; increased complexity can obscure experimental interpretation
**Body-on-a-chip**	Multiple organ/tumor-on-a-chip modules linked together	Relevance for systemic effects between organ systems; permits metastatic TME investigation	Technically challenging arrangement; increased complexity can obscure experimental interpretation

Further, multiple cell types can be introduced to form signaling networks that co-modify their respective behaviours to be reflective of the *in vivo* setting. The relationship between different cell interactions can be augmented by deliberate cellular arrangements by means of 3D bio-printing in order to construct an architecture better reflective of that *in vivo*. Another step in the sophistication of culture models is the incorporation of microfluidic models that help to simulate the effect of local vasculature; continuously refreshing the cultures of nutrients and clearing waste as opposed to more traditional static cultures. Furthermore, microfluidic approaches enable the establishment of well-defined diffusion gradients. Finally, towards the zenith of intricacy for modern models are the body-on-a-chip initiatives whereby models of multiple organs are linked together by microfluidics to provide an insight in systemic signaling and toxicology.

Concomitantly with the increase in cumulative refinement of cell culture techniques is the innovation of organoid models of healthy tissue. These are self-organizing, 3D cultures, generated from a stem cell population that can generate macro-structures of multiple cell types with polarity and arrangements that are extraordinarily similar to the organ from which they were derived (Kim et al., 2020). An in-depth discussion of organoids is beyond the scope of this review but is deftly addressed elsewhere (Fong et al., 2016; Avnet et al., 2019; D'Agosto et al., 2019; Yang et al., 2019). The application of this technology to derive tumor counterparts to organoids of the same organ has also been performed with much success resulting in “tumor organoids” (Baker et al., 2016; Zanoni et al., 2020). Although spheroids and (tumor) organoids are both cultivated in 3D, it is worth remembering that these are not interchangeable terms or technologies (Zanoni et al., 2020). Organoids and their tumor counterpart are derived directly from biopsied materials, contain a stem cell population, and have not undergone culture adaptation in 2D conditions. In contrast, spheroid cultures are typically generated from established cell lines introduced into a low-adherence environment and do not self-organize.

The availability of cell types of the TME, such as fibroblasts, CAFs, immune cells and endothelia has led to attempts to model the TME by means of co-culturing cancer cells together with these other cell lines. Combining these multiple cell types together *in vitro* has made it possible to delineate the interaction between these different cell types and elucidate their contribution to the development of tumor.

In this review we aim to compare and contrast the ever-expanding array of cell culture models used to explore the interactions of the tumor-stroma, with a particular emphasis on co-culture systems. We examine the requirements and major aspects in constructing a biologically relevant co-culture system. In particular, we outline co-culture arrangements and how they have been utilized in tumor-stroma research, followed by examples of how co-culture models have contributed to our knowledge of particular aspects of the TME. Finally, we present a set of guidelines for generating and reporting co-culture models with an emphasis on reproducibility.

## 2 Co-culture models; conception and construction

A co-culture, as the name implies, is the culturing of multiple cell types *in vitro*. Typically, co-cultures are tools to explore how disparate cell types modify their respective behaviours either under ambient conditions or with respect to modulating responses to stimuli such as chemotherapeutics in the case of cancer. Co-cultures can be constructed by multiple means including direct derivation of *ex vivo* tissue explants, or by deliberate combination of derived cell lines, where the complexity of studied interactions and technical sophistication of co-culture construction can both vary (Figure 2; Table 1). Whatever the case, the construction of a co-culture system requires the integration of three key aspects; i) the cell types and interactions to model, ii) their physical arrangement and ECM context and, iii) their media environment. Here, we discuss each of these three conceptual building blocks for constructing a co-culture system.

### 2.1 Cell type characterization and interactions

Arguably, foremost of these in importance is consideration of the cell types, and how many, to include and the type of interaction to examine. Most co-culture models are di-cultures that examine two cell types, however tri-cultures and models incorporating more cell types exist too. Complexity of interactions and analysis exponentially increases with increasing number of cell types, and as such is a limiting factor to the number of cell types examined simultaneously.

The type of interaction between defined cell types can be explored in several ways. Cells co-cultured together in which juxtacrine and paracrine signaling between the different cell types is permitted are referred to as “direct” co-cultures. Paracrine-only interactions can be investigated using “indirect” co-cultures whereby cell types are separated by a physical barrier so that reciprocal communication is possible only *via* secreted factors. An example is in transwell assays whereby use of a sieve-like scaffold inserted into culture dish allows for cells culture both on the culture dish plastic, and also the sieve-like scaffold, where when sharing the same media, may secrete factors that can be reciprocally received without including cell-to-cell contact signaling between different cell types (Öhlund et al., 2017) (Figure 2, “Co-cultures”, right). Both direct and indirect methods are co-cultures, as long as it is possible for cell types to signal reciprocally; consequentially a conditioned media study does not qualify as a co-culture experiment.

There are plenty of examples of co-culture experiments exploring different cell type interactions and in particular those of the tumor-stroma. An exploration of these occurs later after the other conceptual building blocks are considered (See Section 3 “Co-culture models with focus on certain aspects of the tumor microenvironment”).

### 2.2 Physical arrangement of cells and ECM context

**FIGURE 3:**
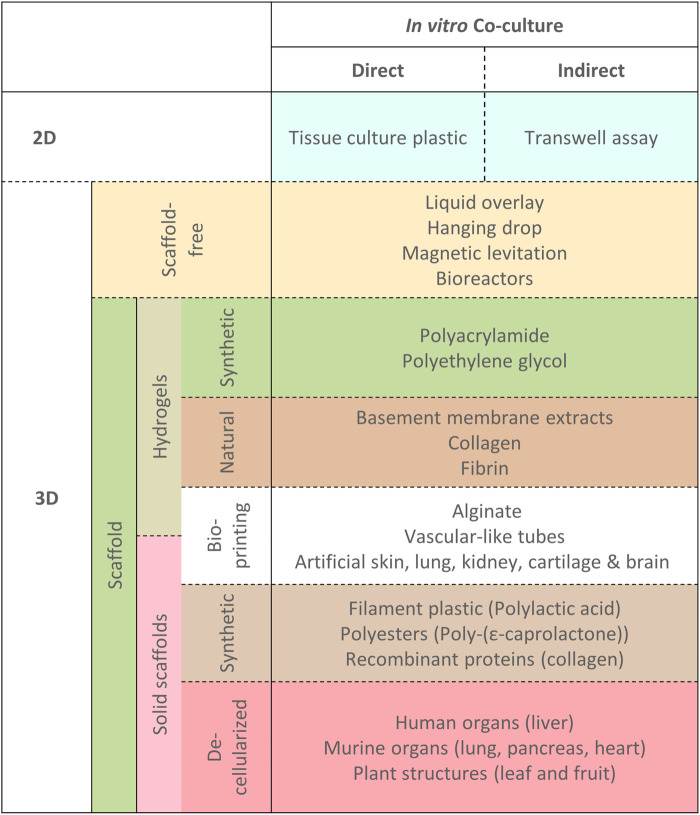
Different physical model types for exploring co-cultures. A hierarchical overview of different physical arrangements of cells for exploring co-cultures with examples as referenced in the text. Dotted lines indicate that techniques/models are not mutually exclusive, where co-culture models may be constructed by combination of any of the approaches outlined.

The next major aspect to consider is the physical arrangement of the cells and consequently what type of model to use. Development of co-culture models of the tumor-stroma has yielded many different approaches which vary with respect to several factors. The two most disparate approaches to acquiring complex co-cultures for experimentation and study are the difference between examining *ex vivo* tumor explants in tissue culture and contrasted with constructed co-cultures with deliberate addition of different cell lines together *in vitro* (Figure 3). Figure 3 describes the general subdivisions of the various cell culture models available and can be used with reference to the following section headings.

Concurrently, the ECM context utilized for the model requires due consideration. The ECM has a profound influence on cell behaviors in terms of mechanotransduction, wherein of mechanical properties such as stiffness or fibrillar alignment are converted into biochemical signals (Humphrey et al., 2014). However, the ECM is not simply just a physical scaffold for cells. In addition, there are a plethora of chemical signals embedded within the ECM which is manifest in terms of specific protein composition such as structural proteins as well as embedded growth factors (Cox and Erler, 2011). These factors are key for determining representative models of cell behaviour and also for examination of ECM remodeling behaviours of co-culture induced ECM remodeling proteins such as matrix metalloproteases (MMPs) and tissue inhibitor of MMPs (TIMPs), where an appropriate ECM context is vital to understand their relevant *in vivo* function (Mott and Werb, 2004; Butcher et al., 2009; Cox and Erler, 2011). MMPs act to remodel the ECM composition and promote its resorption and also act to release embedded factors from the ECM into the TME (Sternlicht and Werb, 2001). There are 23 identified MMPs in humans, with diverse effects, including proliferation, apoptosis, angiogenesis, inflammation, resistance and metastasis. On the other hand, there are four TIMP proteins identified in humans, that act to inhibit MMPs. Generally, all TIMPs overlap in their effects to inhibit MMP activity, although with slightly different efficacies for each MMP, and the balance between MMP and TIMP activity in tissues has often been regarded as crucial in pathology (Visse and Nagase, 2003). A full review of MMPs and TIMPs is beyond the scope of this review, which is covered competently elsewhere (Sternlicht and Werb, 2001; Woessner, 2002; Visse and Nagase, 2003; Kessenbrock et al., 2010; Moore and Crocker, 2012; Arpino et al., 2015; Cabral-Pacheco et al., 2020), however here we will explore some examples that highlight their importance and relationship to the ECM.

MMPs often exert multiple effects that may overlap between MMPs and can be antagonistic even for the same MMP. For example, MMP-1, MMP-3 and MMP-9 exert proinflammatory effects by processing interleukin (IL)-1β from its precursor (Schönbeck et al., 1998), however MMP-1, MMP-2 and MMP-9 also demonstrate anti-inflammatory effects by degrading IL-1β itself (Ito et al., 1996). Further, major MMPs involved in promoting tumor angiogenesis, which is an important step for tumor vascularization and also represents an avenue for metastasis, include MMP-2, MMP-9 and MMP-14. As an example, MMP-9 promotes the bioavailability of sequestered vascular endothelial growth factor (VEGF), a potent inducer of angiogenesis, in pancreatic islets (Bergers et al., 2000). On the other hand, in other ECM contexts, MMP-9 (as well as MMP-3, MMP-7, MMP-13 and MMP-20) acts to generate endostatin, a potent inhibitor of angiogenesis, by cleaving type XVIII collagen in the ECM (Heljasvaara et al., 2005). These examples alone demonstrate that MMP function is dependent on ECM composition and that appropriate ECM context used in assays is crucial to understanding relevant effects on studied cell types.

#### 2.2.1 *Ex vivo* tissue tumor explants

Patient-derived explants (PDE) are generated by direct explant of tumor tissue biopsies into *in vitro* culture conditions and are arguably the most direct method for modelling the *in situ* tumor-stroma *in vitro*. Although they are a heterogeneous admixture of cells of the TME the cell type input is poorly defined and consequently they are distinct from co-cultures constructed to explore specific cellular interactions. There is also difficulty in acquiring whole tissue, especially from patients. Additional issues include the maintenance of whole tissue; in particular preservation of the stem cell niche, sufficient nutrient perfusion and avoiding imbalance of particular cell types is a challenge. As a result, most PDE can only be maintained for up to 72 h (Powley et al., 2020). Reproducibility and determining the most effective readouts to address the particular scientific question are also difficult with limited supply of tissues and where there is the problem of inter-tumoral and inter-patient variation.

Despite these challenges, there is still considerable work in the tumor field that works with *ex vivo* tumor explants where most studies tend to focus on patient-specific drug response in these tumor tissues (Collins et al., 2020; Powley et al., 2020). That said, examples of successful assays do exist, such as in prostate cancer where the effect of therapy on the TME was specifically examined and the phenomena that enzalutamide reinforced the tumor-restrictive desmoplastic stromal pattern in PDE was discovered (Shafi et al., 2018).

Another example is in breast cancer, where slice cultures of tumor explants were exposed to drugs and counterpart material was harvested for gene expression profiles by microarray analysis. Tumor explants that were resistant to Neoadjuvant paclitaxel chemotherapy appeared to have reduced natural killer cell gene signatures, indicating reduced natural killer cell infiltration (Garcia-Chagollan et al., 2018).

PDE culture also allows for precise investigation as to the penetrance of drugs into the tumor itself by mass spectrometry imaging, where a lack of penetrance may be related to the stromal composition. In one study, it was found by mass spectrometry imaging that platinum ions are excluded/depleted in the site of cisplatin resistant non-small cell lung cancer cells, but present instead in the surrounding stromal tissue and that this exclusion/depletion was the reason for cisplatin resistance in these tumors (Karekla et al., 2017).

#### 2.2.2 *In vitro* co-culture models

Approaches to co-culture *in vitro* involve both 2D and 3D systems, although the ability to examine cell arrangement and juxtacrine interactions is greatly enhanced by the ability of cells to interact over the surface area of cells made available in 3D conditions. In 2D, cells are cultured in a monolayer, often on tissue-culture treated plastic, or on semi-permeable membranes. Cell culture plastic includes the confounder of signaling mediated by its mechanical properties, adding unnecessary artefacts to data interpretation. Moreover, 2D cultures are often faced with the problem of extreme selection pressure of the most proliferative cells that induces genomic instability, an issue that 3D cultures appear to avoid (Kaushik et al., 2018).

When it comes to 3D culture systems there are broadly two main approaches; scaffold or scaffold-free. Scaffold-free approaches rely on methods to prevent cells from attaching to cell culture plastic, resulting in multi-cellular spheroid aggregates and include techniques such as liquid overlay, hanging drop, bioreactor/agitation and magnetic levitation. Scaffold approaches include the use of porous, dried, solid macrostructures that cells colonize and hydrogel cell-suspension approaches. Scaffolds can be produced synthetically or derived from natural sources which are processed to be made suitable for cell colonization. Another feature of scaffolds is their compatibility with bioprinting approaches which facilitate precise orchestration of cellular and matrix arrangements.

#### 2.2.3 Scaffold-free approaches

Scaffold-free approaches are arguably the simplest approach to *in vitro* cell culture, and all rely upon preventing cells from adhering to culture plastic.

##### 2.2.3.1 Liquid overlay technique

This method is one of the more cost-effective approaches to generate 3D cultures and co-cultures, whereby culture plastics are coated with a thin layer of relatively inert substances such as agar, agarose, or more complex substrates such as Matrigel or de-epidermized acellular dermis to prevent cell cultures from adhering directly to the plastic. Instead, the cells aggregate to form spheroids. It is possible, in the constitution of these models, to co-culture multiple cell lines together and examine the resulting interactions in both healthy and tumor models (Colley et al., 2011; Metzger et al., 2011; Yakavets et al., 2019). Commercially, ultra-low attachment plates have been developed which are coated with a hydrophobic polymer that inhibits cell adhesion to the culture plastic without the need to coat with alternative substrates.

##### 2.2.3.2 Hanging drop method

The hanging drop technique is used to generate 3D multicellular spheroids by seeding cells in culture medium onto the inside of a Petri-dish lid. When inverted, this leads to the formation of a hanging droplet, which is stabilized by surface tension, containing cells which are unable to attach to the air-liquid interface and instead aggregate together and form spheroids (Kelm et al., 2003). The size of spheroids desired is rather robustly controllable by the initial seeding cell number used, and techniques for co-culturing 3D spheroids by hanging drop are also developed (Kelm et al., 2003; Foty, 2011). A major benefit of this technique is that any ECM that forms in these droplets is purely generated by the cells themselves, and their behaviour is not susceptible to potentially artefactual response to foreign ECM. Conversely, this benefit could also be viewed as a disadvantage since the arrangement of these cells is informed in an environment initially devoid of ECM which may too affect the overall development of the spheroid. Another necessary drawback of this method is that it is limited in volume by the surface tension of the droplet, meaning that it is difficult to exceed around 50 μL of culture per drop and further, the physical arrangement can make medium exchange without disturbing the spheroids challenging (Kurosawa, 2007; Mehta et al., 2012).

A conceptual variant of the hanging drop technique involves the use of magnets to suspend cells that have accumulated magnetic nanoparticles within cell culture media and away from direct contact with any plastic interface. Surrounded by media, these cells aggregate and form spheroids (Lewis et al., 2017). This approach does not have the same limitations of culture volume that the hanging drop technique must be bound by. Indeed, it has also been used in the case of studying tumor-stroma in breast cancer co-culture models (Jaganathan et al., 2014). Introduction of magnetic beads into various cell types have demonstrated that they do not affect cellular proliferation nor induce an inflammatory response (Haisler et al., 2013; Tseng et al., 2013), however, there are concerns that the use of high concentrations magnetic beads can have toxic side effects on cells (Nath and Devi, 2016). Further, depending on the assay employed, the presence of iron oxide in culture media can cause a discoloration that can interfere with colorimetric assays (Haisler et al., 2013). There is also the possibility of potential compatibility issues with other downstream assays based on magnetic principles.

##### 2.2.3.3 Bioreactors

Bioreactor based 3D cell culture primarily focuses on upscaling cell culture production; and generates vast quantities of spheroids in a robustly controllable manner and at scales suitable for industrial purposes, such as harvesting products of metabolism such as growth factors or antibodies (Lichtenberg et al., 2005; Guller et al., 2016). The underlying principle in bioreactors is to prevent cell adhesion to culture vessels by the introduction of constant agitation *via* one of several mechanisms such as stirring, chamber rotation, or fluid pumping.

Despite the general use of bioreactors for scalable industrial/commercial purposes, they have been used in the maintenance of tumor co-cultures (Lichtenberg et al., 2005; Guller et al., 2016). For example, stirring bioreactors have been used for the preparation of spheroids containing head and neck squamous carcinoma cells co-cultured with peripheral blood mononuclear cells as a model for micro-metastases (Hirschhaeuser et al., 2009). The effect of catumaxomab, a monoclonal antibody used as a chemotherapeutic against malignant ascites, was adequately recapitulated in this model system. An alternative co-culture example used a rotary bioreactor to co-culture undifferentiated colon adenocarcinoma cells together with normal fibroblasts on microcarrier beads (Goodwin et al., 1992). Here the effect of co-culture was very different to that of mono-cultured colon adenocarcinoma cells, which produced spheroids demonstrating minimal differentiation. However, when co-cultured with normal fibroblasts, the colon adenocarcinoma cells, initial growth was limited to only the fibroblast population until they covered the microcarrier beads. Then the cancer cells proliferated at an accelerated rate, forming tissue structures up to 1.5 cm in diameter containing glandular structures, apical and internal glandular microvilli, cellular polarity and organization similar to normal colon crypt development (Goodwin et al., 1992). Much more recently, perfusion-based bioreactors have been used for maintaining co-cultures of breast cancer cells co-cultured with fibroblasts which also report considerable cell growth that exceeds that of the static co-culture counterpart (Nguyen et al., 2022).

#### 2.2.4 Scaffold approaches

Scaffold approaches rely upon providing a medium within which cells are physically supported and able to proliferate in 3D. There are two main approaches to scaffolding techniques; hydrogels, which provide a relatively homogeneous physical medium that supports cell growth and autonomous organoid morphology, and solid scaffolds, which are porous structures within which cells attach but are capable of 3D exploration through the physical scaffold and arrange therein.

##### 2.2.4.1 Hydrogels

The use of hydrogels, networks of cross-linked polymeric material, is an alternative approach to 3D culture and co-culture (Caliari and Burdick, 2016; Yazdi et al., 2020). Hydrogels are a gel-like material that are usually composed of hydrophilic polymers that are water-absorbing, yet water insoluble, and consequently present a 3D context in which cell cultures may be encapsulated whilst permitting sufficient nutrient and cellular waste circulation. Hydrogels can be naturally or synthetically derived.

A feature of this approach is the mechanical resemblance of hydrogels to natural ECM since they exhibit a soft, tissue-like stiffness that is composed of natural polymers such as collagen, fibrin or alginate or synthetic polymers such as polyacrylamide or polyethylene glycol (Lutolf and Hubbell, 2005; Miroshnikova et al., 2011; Caliari and Burdick, 2016). The choice of polymer is important since some, such as alginate and polyethylene glycol, are relatively inert, whilst others can contribute directly to cell signaling, such as fibrin or collagen (El-Sherbiny and Yacoub, 2013; Caliari and Burdick, 2016). Indeed, the ECM protein composition of hydrogels is customizable; enabling the ability, conceptually, to tailor hydrogel composition to replicate those of context-appropriate niches (Nicodemus and Bryant, 2008). In practice, however, this remains challenging since it is difficult to perfectly recapitulate the ECM compartment of a target tissue with the precise admixture of all ECM proteins and growth factors artificially (Bonnans et al., 2014; Yue, 2014).

Combining hydrogels with reconstituted basement membrane extracts is an approach that makes a compromise between the challenging generation of perfectly tailored hydrogel mixtures by artificially complementing the hydrogel with ECM proteins and growth factors, and the utilization of a more biochemically inert environment. One of the best known commercially available products, Matrigel (Corning), is derived from basement membrane extracts of Engelbreth-Holm-Swarm mouse sarcoma, although other competitor products such as Geltrex (Thermo) or Cultrex (R&D systems) take a similar approach (Kibbey, 1994; Kleinman, 2001). Whilst this method does have the advantage of providing a more relevant biological complement of ECM proteins in the hydrogel, there are some drawbacks such as that the material is necessarily not tailored for specific microenvironments. An additional drawback is the requirement for animal sacrifice, which introduces corresponding batch-to-batch variability and processing expense, despite the controlled fabrication process, as well as difficulties for approval for potential therapeutics whose production relies on such material derived from animal sarcomas (Polykandriotis et al., 2008). That said, there are commercially available products, such as VitroGel Hydrogel (TheWell Bioscience), an animal origin-free polysaccharide hydrogel that is entirely synthetically derived and can be modified with xeno-free bio-active ligands for various organoid applications, which may overcome issues associated with xeno-derived materials (Huang, 2019). Derivation of *in vitro* sources of ECM from fibroblasts area also developed, providing another means by which to acquire cell-derived ECM proteins from an *in vitro* context (Serebriiskii et al., 2008).

Overall, though, hydrogels are employed prominently in 3D cultures and co-cultures due to their structural similarities to natural ECM and versatility in application. Indeed, they have been used extensively in co-culture applications in a number of ways, including direct cell mixture and seeding, as well as more creative approaches to examine cell invasion and signaling.

##### 2.2.4.2 Solid scaffolds

Other approaches to 3D culture are based upon providing suitable physical niches for cells to adhere to and proliferate within (Chan and Leong, 2008). Solid scaffolds, unlike hydrogels, are solid macromolecular structures that provide solid attachment points for cells, rather than a free-form gel without solid attachment points. Scaffolds can be generated in a variety of different ways from a variety of substrates and so consequently there is a large degree of variation in the porosity, permeability, chemistry and mechanical characteristics in different scaffolds (Sieh et al., 2010; Talukdar et al., 2011; Fong et al., 2013; Dunne et al., 2014; Lu et al., 2014; Sieh et al., 2014; Stratmann et al., 2014; Villasante et al., 2014). The aim, overall, is to mimic the physicochemical properties of the *in vivo* counterpart to the cell culture model. Broadly, scaffolds are either generated synthetically from defined components, or are derived from natural tissue that is then decellularized; removing the native cells to provide a scaffold for the desired cell culture to colonize.

A reproducible way to generate solid scaffolds is to produce them synthetically from defined components. There are many different biopolymers that have been used as the primary scaffold constituents such as plant derived recombinant human collagen (Willard et al., 2013), alginate (Lee and Mooney, 2012), polylactic acid (Serra et al., 2013) and poly-(ε-caprolactone) (Sieh et al., 2010; Fong et al., 2013; Li et al., 2017). The easiest way to generate a porous scaffold is to freeze dry the material once polymerized. In this way, the water in the scaffolds is sublimated and thus removed without collapsing the polymerized structure to leave a porous material. By manipulating various factors such as the polymer solution water content and freezing temperature during the freeze-drying process, different porosities, including pore dimension can be altered as can biodegradation rates be altered with respect to the polymer molecular weight.

An alternative method of solid scaffold generation is to take directly tissue or organs with an appropriate structure, remove the endemic cells and then re-seed with the cell type of interest (Crapo et al., 2011; He and Callanan, 2013; Rijal and Li, 2017). The objective of decellularization is the removal of all cellular material without adversely affecting the mechanical and signaling properties of the remaining ECM. The advantage of such an approach is that the relevant conditions for each organ with respect to the mechano-chemical properties of the ECM is naturally accounted for, providing the appropriate niche for study. Examples include mouse lung (Crabbé et al., 2015), mouse pancreas (Guruswamy Damodaran and Vermette, 2018), rat heart (Robertson et al., 2014) and human liver (Mazza et al., 2015).

One drawback of decellularized tissue scaffolds is the availability of relevant tissue/organs which are usually animal-derived, although in the field of regenerative medicine, human donor organs are also used which also require appropriate ethical considerations. Some groups have, however, generated decellularized tissue scaffolds derived from non-animal sources such as plant-scaffolds, examples include decellularized apples and spinach leaf, as well as bacterially-derived cellulose and, potentially, fungal-derived chitin, reviewed elsewhere (Carletti et al., 2011; Jayakumar et al., 2011; Huber et al., 2012; Mallick and Cox, 2013; Modulevsky et al., 2014; Derakhshanfar et al., 2018; Campuzano and Pelling, 2019; Dikici et al., 2019; Hickey and Pelling, 2019). These approaches yield 3D structures that can be used for mammalian cell culture, but of course lack many relevant signaling molecules found in mammalian ECM. In the case of decellularized plant-tissue scaffolds, they can be coated with biologically relevant proteins for different cell types.

##### 2.2.4.3 Bioprinting

Scaffold approaches, both solid and hydrogel, are readily amenable to 3D bioprinting technology (Rider et al., 2018; Turnbull et al., 2018; Nikolova and Chavali, 2019; Mancha Sánchez et al., 2020). 3D printing refers, in general, to the construction of customized designs from materials printed out, solidified and bound together under computational control. The ability to print in 3D using relatively simple materials has been important in the industrial sector at large, but also can be adapted for use in a cancer context, which is reviewed in depth excellently elsewhere (Datta et al., 2020; Kang et al., 2020; Augustine et al., 2021).

As a brief overview, 3D bioprinting is defined by Datta et al. (2020) as the layer-by-layer deposition of bio-inks and cells in a spatially defined manner to generate viable 3D constructs. Bio-inks is a broad term that includes printable scaffold materials that may or may not already contain cells such as alginate, decellularized ECM, microcarriers and hydrogels, as well as including printable cell or tissue structures such as spheroids, cell pellets or organoids. The 3D printing construction is conducted by computer-aided design to generate layered models that are more accurate spatial reconstructions of the *in situ* setting. Alternatively, 3D bioprinting approaches could in principle be used to test hypotheses by constructing custom layered designs that specifically model an arrangement that is not naturally found *in vivo*. 3D bioprinting consequently allows for a considerable degree of experimental design and flexibility. There are already 3D bioprinted systems to recapitulate vascular-like tubes (Yu et al., 2013), artificial skin (Lee et al., 2014), lung (Murphy and Atala, 2014; Marrazzo et al., 2016), kidney (Lawlor et al., 2020), cartilage (Cui et al., 2012) and brain (Han and Hsu, 2017). Indeed, cancer specific bioprinted models exist for breast (Wang et al., 2018), brain (Heinrich et al., 2019), ovarian (Baka et al., 2022) and skin (Browning et al., 2020) cancers, among others.

A resultant property of a custom designed, printed, structure is the ability to alter the mechanical properties of the model by appropriate layering. For example, layered of polyethylene glycol structures of different stiffnesses revealed that the third dimension was crucial for understanding cell motility (Soman et al., 2012). This was further corroborated by another study that developed bioprinted hydrogel matrices with stiffness gradients to mimic the physiochemical properties of the TME to study cancer cell migration (Gupta et al., 2015). However, stiffness is not the only property that is tunable in bioprinted systems. For instance, the spatial distribution and temporal release of embedded biochemical factors in the model can also be specified (Gupta et al., 2015).

In addition to spatiotemporal control of the ECM in bioprinted models, it is possible to incorporate additional cell types and macrostructures into these models. For example, there are many examples of bioprinted models of vascularized tissue, reviewed elsewhere (Datta et al., 2017), and these can be constructed to also incorporate cultures of cancer cells (Meng et al., 2019; Cui et al., 2020). Indeed, these models can also be coupled with microfluidic approaches to examine the effect of fluid dynamics and media exchange on bioprinted cultures (see Section 2.3.1 “Microfluidic technologies”). In one example in a bioprinted model, it was demonstrated that shear stress can induce tumor cell G2/M cell cycle arrest from 12 dyn/cm^2^ whereas G0/G1 cell cycle arrest occurred in cultures at 0 dyn/cm^2^ (Chang et al., 2008).

Overall, bioprinted models offer considerable improvements with respect to precise and reproducible tissue mimicry, however they require considerable optimization in terms of material properties, including scaffold density and biocompatibility, as well as potential for toxicity from products of scaffold degradation.

### 2.3 Media; selection and exchange

Another key aspect is the selection of the most appropriate media for the co-culture. For many monoculture cell lines, media is tailored towards the sustained propagation of that particular cell type. This is particularly true for organoid and tumor organoid cultures where the media is designed for the propagation of the epithelial stem cell type and its progeny and to inhibit the growth of other cells types such as fibroblasts (Sato et al., 2009; Drost et al., 2015; Fujii et al., 2016; Seino et al., 2018). Whilst useful for culturing organoids alone, co-culture experiments examining the interactions between organoids and fibroblasts could lead to erroneous conclusions when cultured in a fibroblast-inhibiting organoid feed medium (Sato et al., 2009; Öhlund et al., 2017). Furthermore, the context of cell signaling is informed by the environment within which the cells are situated. Therefore, careful characterization of the media is important for the interpretation of interactions identified *in vitro*.

In many co-culture experiments, the media used for the monoculture of one cell line or another is used, or sometimes mixtures of the respective monoculture media are utilized (Dudás et al., 2011a; Dudás et al., 2011b; Goers et al., 2014; Chaddad et al., 2017; Mazio et al., 2018). Whilst there is no definitive answer to the optimal medium to use for co-cultures, some steps could be taken to ensure an appropriate selection is made. Ideally, a media composition that is reflective of the organ or TME is ideal, however this is usually not readily available. As an alternative preparation, one could switch the respective monoculture media to ensure each cell line is sustained, which would indicate the lines are well suited to co-culture in the same media. Further, a systematic removal of supplemented growth factors/components can be performed in order to attain the least complicated media that is able to sustain all cell lines. Alternatively, if cultures are maintained ordinarily in monoculture in different media, it may be possible to gradually adapt cell cultures to a different medium that all cells to be co-cultured could tolerate (Bidarra et al., 2011). However, this would then require the validation of the input cells in these new conditions. If a symbiotic interaction between co-cultured lines is expected, then using media with minimal growth factor supplementation can act to maximize the effects of symbiotic interactions between cell lines. Alternatively, an approach that is sometimes used is the application of conditioned media from relevant cell lines, which has the advantage of containing factors anticipated to be relevant but has the drawback of batch to batch (including lab to lab) variability that can hamper reproducibility.

When it comes to co-culture media, another important consideration is whether the model will utilize a “static” media approach, where media is added at the beginning of an experiment and left or fully exchanged every few days. Alternatively, a “dynamic” arrangement is possible by means of microfluidics whereby cultures are supplied with media continuously or semi-continuously.

#### 2.3.1 Microfluidic technologies

Microfluidic approaches enable the modelling of continuous media replenishment and waste exchange as well as the influence of fluidic pressure on cell behaviour. Such approaches have been implemented in a number of ways and has also been integrated into the development of organ-on-a-chip and tumor-on-a-chip devices (Piotrowski-Daspit et al., 2017). The next extension of organ/tumor-on-a-chip technologies is the combination of multiple organ/tumor-on-a-chip modules together to mimic a more systemic approach to researching tumor biology (Figure 2) (Sato et al., 2009; Skardal et al., 2016; Edington et al., 2018; Sontheimer-Phelps et al., 2019).

Microfluidics offers the possibility to study tumor models in a more dynamic environment that can respond to additional cues such as controllable fluidic pressure, the physical characteristics of flow and models of endocrine interactions. Given the fluidic element incorporated into these models that acts as a surrogate for vasculature, microfluidic devices have enabled more detailed research into the processes of metastasis, including cellular delamination from the primary tumor site and requirements for circulating tumor cell adhesion to a new site. By using microfluidic techniques, even 2D models have been used to provide insight into tumor biology in co-culture and metastatic potential (Li W. et al., 2018). This is particularly true for paracrine interactions, where different cell types are kept physically separated, but able to signal to each other *via* secreted factors.

Microfluidics researchers have also developed models that utilize 3D culture. An early 3D example in the tumor context utilized tumor spheroids derived from a malignant pleural mesothelioma cell line maintained in a “multi-S-shaped” microfluidic channel. In this example, spheroids were more resistant to cisplatin than spheroids in static (non-microfluidic) culture (Ruppen et al., 2014). The extension into 3D co-culture models have also been developed; Yongli Chen *et al.* (2018), for example, developed a breast cancer model under flow that mimicked vasculature using a monolayer of human umbilical vein endothelial cells (HUVECs), contained ECM hydrogel derived from basement membrane extract, and multicellular tumor spheroids representative of triple-negative breast cancer (TNBC) and non-TNBC (Chen Y. et al., 2018). In this model, the efficacy of doxorubicin (a drug used in the treatment of breast cancer, amongst others) delivery on carbon nano-particles across the endothelial membrane into tumor spheroids was monitored.

More complicated co-culture examples in microfluidic devices can be considered tumor-on-a-chip devices, although the distinction between a tumor-co-culture in a microfluidic setting and a tumor-on-a-chip is blurred (Trujillo-de Santiago et al., 2019). Regardless, organ/tumor-on-a-chip systems are developed as an advance on microfluidic techniques that also combine several over aspects of tissue engineering. These devices act to recapitulate organ-specific functions in self-contained units and as the technology becomes more advanced, several “organ” chips can be linked together to generate more advanced models of multi-organ, systemic, behaviour termed “body-on-a-chip” devices.

These systems can be constructed in several ways, including those already described such as bioprinting. A relatively simple tumor-on-a-chip co-culture model was developed to study the progression from ductal carcinoma *in situ* to invasive ductal carcinoma in the breast cancer context (Sung et al., 2011). In this model, non-tumor human mammary fibroblasts were co-cultured with mammary epithelial ductal carcinoma *in situ* (DCIS) cells and maintained in a hydrogel consisting of a Matrigel and collagen type I mixture in a microfluidic device. In response to co-culture with non-tumor fibroblasts, tumor cells adopted a different morphology which altered with respect to their distance from the normal fibroblast interface. This change in morphology was reminiscent of the transition to invasive ductal carcinoma and there also appeared to be a biphasic effect, whereby paracrine signaling could initiate a change in morphology in the tumor cells up to 1.5 mm away, but the transition to an invasive phenotype appeared completed upon cell-cell contact between tumor and non-tumor cells.

Another tumor-on-a-chip example in the breast cancer context examined malignant epithelial cell invasion into healthy stroma. In this model, the two cell types were cultured in two concentric chambers; stromal fibroblasts (either normal or cancer associated) in one chamber, and the malignant epithelial breast cancer cell line MCF7 in the other chamber (Gioiella et al., 2016). Normal fibroblasts, when co-cultured with MCF7 cells were induced to expression of alpha smooth muscle actin (α-SMA) and platelet derived growth factor (PDGF) which are markers of myofibroblastic activation, and correspondingly generated a remodeled ECM composition *via* elevated MMP-9 production as well as production of fibronectin, hyaluronic acid and collagen that is also seen in the TME *in situ*.

A different study examined reactive oxygen species concentrations in necrotic cores of glioblastoma and colon carcinoma cells with and without co-culture of natural killer cells introduced *via* the fluidic system (Ayuso et al., 2016). Tumor cells were cultured in a hydrogel in a central chamber flanked by two lateral micro-channels through which flow was maintained for the provision of media, oxygen, natural killer cells and tumor-specific chemotherapeutics (Doxorubicin for colon-cancer and Temozolomide for glioblastoma). The growth rates of the tumor cells were examined with respect to their distance from the microchannels and demonstrated that the chemotherapeutics exhibited a differential effect on the tumor cells with greater efficacy against cells in the hypoxic core of the model(s). In this model, natural killer cells migrated into the central chamber and penetrated into the tumor cell culture.

Another research group constructed tumor-on-a-chip devices in the pancreatic cancer context using patient-derived-organoids co-cultured with commercially available pancreatic stellate cell and monocytic lines (Haque et al., 2022). In this case, the patient derived tumor organoids were better sustained by the inclusion of pancreatic stellate cells and monocytes into the culture. The system was also amenable to examine the efficacy of chemotherapeutic agents, which showed differing effectiveness against the tumor organoids dependent on the inclusion of the stromal cell types. Ultimately, this platform could be used for examining or predicting patient-specific responses to chemotherapeutics (Haque et al., 2022).

Ultimately, the combination of multiple organ/tumor-on-a-chip modules together constitute a “body-on-a-chip” system (Miranda et al., 2018). These systems can be used to mimic a systemic response to tumor activity, metastasis, or systemic drug activity and biodistribution (Miranda et al., 2018; Wang et al., 2020). For example, Frey *et al.* (2014), designed a system whereby colorectal cancer spheroids (generated by hanging drop) are microfluidically linked to hepatic spheroids (Frey et al., 2014). In this system, chemotherapeutics against the colorectal cancer cells are first exposed to the hepatic spheroids, which metabolize the drug prior to its subsequent exposure in flow to the colorectal cancer spheroids and maintained in a closed loop. This rather cleverly mimics the situation of drug metabolism *in vivo* which is often metabolized by the liver prior to reaching the target site of action, whereby its effects are altered. In this study, they observed that the anti-cancer prodrug cyclophosphamide did not affect colorectal cancer cell growth unless the compound was first metabolized by hepatic spheroids. A parallel experiment utilizing conditioned media exchanges to simulate a static experimental design switched media from hepatic cells treated with cyclophosphamide to colorectal cancer cells cultures did not demonstrate the antitumor effect of the metabolized cyclophosphamide.

## 3 Co-culture models with focus on certain aspects of the TME

### 3.1 Cancer associated fibroblasts

Fibroblasts are non-epithelial, non-immune cells without any association with a basement membrane within the interstitial space embedded within the ECM (Tarin and Croft, 1969). In normal tissue, fibroblasts generally display little transcriptional or metabolic activity and typically described as quiescent or resting (Kalluri, 2016). However, fibroblasts may become activated and contribute to functions of wound healing, as well as chronic or acute inflammation (Gabbiani et al., 1971; Micallef et al., 2012). They are probably best defined as a resting mesenchymal cell with the potential to become activated to function like a mesenchymal stem cell (Kalluri, 2016). It is also pertinent to note that so far there are no known markers unique to activated fibroblasts, nor any that have complete coverage of all activated fibroblasts. That said, common markers used to identify myofibroblasts, a major subtype of activated fibroblasts include fibroblast activation protein and α-SMA (Sunami et al., 2020).

Many cancers display a prominent desmoplastic reaction resulting in a considerable complement of activated fibroblasts (Chitturi et al., 2015). The function of these fibroblasts share some similarities but also prominent differences with the functions of myofibroblasts in wound healing (Kalluri, 2016). The cancer associated fibroblasts (CAFs) are a heterogeneous collection of subtypes with disparate functions and spatial organization in the TME. Their function is complex and multifaceted; in some cases, promoting tumor progression whereas some functions act to restrain the tumor (Alkasalias et al., 2018; LeBleu and Kalluri, 2018). CAFs are induced to activation by growth factors released by the tumor cells themselves as well as those released by infiltrating immune cells (Alkasalias et al., 2018; LeBleu and Kalluri, 2018). That said, the activation pathways of all CAF subtypes are not yet fully understood, especially in the light of the relatively recent discovery of the multiple different CAF subtypes, which are still being discovered (Sidaway, 2018).

Cancer cell-CAF co-cultures represent a useful tool by which to discover and interrogate the formation and signaling between cancer cells and CAFs. Indeed, many co-culture experiments exploring CAF function have identified that cancer cell-CAF co-culture enhances tumorigenesis compared to tumor monoculture or even with normal fibroblasts (reviewed here and examples to follow) (Liu et al., 2019). For example, when pancreatic stellate cells, a major source of CAFs in pancreatic ductal adenocarcinoma (PDAC), are co-cultured together with PDAC cancer cells on low-adherence plates, more tumor spheroids are formed compared to in monoculture. The expression of the epithelial-mesenchymal-transition (EMT) gene Snail-1 and other genes associated with a cancer stem cell phenotype (ABCG2, Nestin, LIN28) are also upregulated in these cells (Hamada et al., 2012). Although that study did not detect any improvement in tumor cell proliferation, other studies examining the effect of pancreatic stellate cell-derived conditioned media discovered an increase in tumor cell proliferation compared to tumor cells cultured in serum-free media (Hwang et al., 2008). In the tumor organoid context, co-culture with pancreatic stellate cells facilitates enhanced tumor organoid viability for much longer in media devoid of the growth factors usually required to maintain their growth in monoculture. Furthermore, CAFs appear to have roles in conferring resistance to therapeutics. A study examining chemoresistance in PDAC tumor cells co-cultured with CAFs on a Matrigel scaffold determined that CAFs conferred treatment resistance to oxaliplatin and benzoporphyrin derivative photodynamic treatment (Broekgaarden et al., 2019).

Further, the direct co-culture of murine pancreatic stellate cells, together with pancreatic tumor organoids derived from a well credentialed genetically engineered mouse model of PDAC directly led to the discovery of CAF subtypes in PDAC (Öhlund et al., 2017). These initially identified subtypes include myofibroblastic CAFs (myCAFs) and an inflammatory CAF (iCAF) subtype. Here, myCAFs are α-SMA positive, elongated fibroblast-like cells that are found adjacent to tumor cells and upregulate ECM production. In contrast, iCAFs are not found directly adjacent to tumor ducts, rather they are deeper in the surrounding stroma. They appear to be primarily induced by activation of JAK-STAT signaling *via* IL1 signaling and secrete inflammatory cytokines such as IL6 and leukemia inhibitory factor (LIF) that potently promote tumor organoid proliferation (Öhlund et al., 2017; Biffi et al., 2019). In conjunction with indirect co-culture studies, it was also identified that myCAFs require both TGFβ signaling and some juxtacrine signaling with tumor cells, since when kept separate in indirect co-culture transwell assays, myCAFs do not form (Öhlund et al., 2017).

These findings are corroborated in the context of breast cancer, where CAFs also reciprocally signal with tumor cells. Indeed, breast cancer cell lines indirectly co-cultured with CAFs demonstrate upregulated transcription of many genes associated with tumor progression in transwell assays, expressing more transcripts for fibroblast growth factors (FGF2, FGF7), transforming growth factor beta (TGFβ), platelet derived growth factors (PDGFA, PDGFB), inflammatory cytokines (IL6, IL8), ECM remodeling enzymes (TIMP1, MMP11, urokinase plasminogen actovator) and the angiogenic factor VEGFA (Eiro et al., 2018). Additional factors include the promotion of a metastatic phenotype, for example, 2D direct xeno-co-cultures of breast cancer cells with mouse embryonic fibroblasts also helped identify a key role for heat-shock-factor-1 (HSF1) in malignant progression (Scherz-Shouval et al., 2014). However, it is clear that CAFs are not a homogeneous population in breast cancer. At least four CAF subtypes have been reported based upon characterization by flow cytometry and demonstrate different behaviours, including one subtype that has a strong association with immunosuppression (Costa et al., 2018).

In the context of non-small-cell-lung cancer, studies where hanging drop tumor spheroids are co-cultured with fibroblasts identified that fibroblasts acted to stabilize the co-cultured spheroid compared to monoculture attempts of tumor spheroid formation (Nakamura et al., 2019). Further, when co-cultured with fibroblasts overexpressing podoplanin, the proliferation rate of tumor cells in the mixed spheroids was increased but not with podoplanin negative fibroblasts (Nakamura et al., 2019). A different, hydrogel based, spheroid model of squamous cell carcinoma of the lung also identified a modulating effect on tumor cells when co-cultured with CAFs, inducing a more plastic and invasive phenotype in tumor spheroids especially when spheroids were proximal to CAFs (Chen S. et al., 2018). In this same model, however, CAFs acted to restrain the dysplasia seen in tumor cells that expressed high levels of the oncogenic transcription factor SOX2, indicating a multifaceted role of CAFs in tumor progression (Chen S. et al., 2018). Microfluidic models of indirect co-cultures of lung tumor cell lines with normal fibroblasts separated by a semipermeable membrane detected an increase in the fibroblast activation markers α-SMA and vimentin. The tumor cells adopted a more invasive and motile phenotype, with a directional bias towards the fibroblast populations and appeared to be mediated by activated fibroblast GRP78 expression (Yu et al., 2016).

Fibroblasts are critical to maintaining a normal ECM composition, however in response to cancer cell signaling acquire an activated phenotype, becoming α-SMA and fibroblast activation protein positive (Cheng et al., 2005). Breast CAFs produce pro-tumorigenic factors that contribute to fibrosis and ECM remodeling, in particular that of collagen, of the tumor (Corsa et al., 2016). That said, in a study comparing two different 3D ECM backgrounds, ECM production and remodeling by fibroblasts occurred when cultured in Collagen type I but not when in Matrigel, with implications for ECM composition both *in vivo* and for appropriate modelling *in vitro* (Corsa et al., 2016). Further, an alternative study demonstrated that fibroblasts deficient for Hic-5 expression produced more disorganized fibronectin fibers in the ECM, which hindered tumor cell migration compared to their movement on organized fibronectin fibers (Goreczny et al., 2017).

Taken together, cancer cell-CAF co-culture are demonstrably useful to examine the multifaceted roles of CAFs, and their subtypes, in tumor progression and restraint. In the future, these co-culture platforms could allow for identification of novel CAF subtypes as well as specific interrogation of their formation and subtype-specific behaviours, such as ECM deposition, that could be avenues to therapy.

### 3.2 Immune cells

Immune cells have an important role in tumor progression, tumor associated macrophages, for instance can contribute to up to 50% of breast tumor mass (Obeid et al., 2013; Lewis et al., 2017). There are many immune cells present within the TME including lymphocytes (B and T cells), Natural killer cells, macrophages, and dendritic cells, amongst others (Whiteside, 2006; Gonzalez et al., 2018; Torphy et al., 2020). As with other TME reconstitution models that incorporate other cell types with tumor organoid models, diverse components of the immune system can be combined in co-cultures with cancer cells (Yuki et al., 2020).

In tumor organoids derived from a murine model for breast cancer, the addition of tumor associated macrophages isolated from mammary carcinomas induced an invasive phenotype (DeNardo et al., 2009). This invasive phenotype could be inhibited by the addition of M1-type cytokines to these co-cultures (DeNardo et al., 2009). Further, a co-culture of tumor organoids, tumor associated macrophages and CD4^+^ T cells even further induced an invasive phenotype that appears dependent on IL-4 signaling (DeNardo et al., 2009). A different example using patient derived PDAC tumor organoids co-cultured with patient-matched CAFs encapsulated in Matrigel and supplemented with peripheral blood lymphocytes observed the formation of myCAFs as well as lymphocyte infiltration into Matrigel domes that contained the tumor organoid co-cultures (Tsai et al., 2018). In contrast, lymphocytes did not infiltrate into empty Matrigel domes but their infiltrative capacity into control (non-tumor) organoid-containing Matrigel domes was not investigated (Tsai et al., 2018). A different experiment that performed a direct co-culture of colorectal tumor spheroids with T cells and natural killer cells acquired from healthy donors similarly identified that co-culture models could examine immune cell response to the tumor spheroids and both immune cell types rapidly infiltrated the spheroids and potently induced apoptosis of the cancer cells (Courau et al., 2019).

Another co-culture set-up examined cytotoxic T cell activity when co-cultured with dendritic cells that were treated with conditioned media from normal, or tumor, murine gastric organoids (Chakrabarti et al., 2018). Cytotoxic T cells upregulated expression of the receptor for programmed cell death ligand (PD-L1), programmed cell death protein 1, as well as Interferon gamma (IFN-γ) and IL-2 when co-cultured with dendritic cells cultured in conditioned media from tumor organoids, but not their normal counterpart (Chakrabarti et al., 2018). Further, when conditioned media pre-treated dendritic cells were implemented in a tri-culture of cytotoxic T cells together with normal or tumor organoids, cytotoxic T cells migrated to surround tumor organoids (Chakrabarti et al., 2018). The tumor organoids produce PD-L1, and when tri-cultures were also treated with PD-L1 inhibitor, cytotoxic T cells potently induced apoptosis in the tumor organoids (Chakrabarti et al., 2018). These CAF subtypes have been corroborated *in vivo* by transcriptomic studies of patient samples and murine models which also identified the additional antigen-presenting CAF (apCAF) subtype that expresses major histocompatibility complex class II genes and induces T-cell receptor ligation in CD4^+^ T cells (Elyada et al., 2019). To add to the complexity, it appears that these CAF subtypes are plastic and can be induced to form the other subtypes under the right conditions (Öhlund et al., 2017).

In another example interrogating the immune system in mouse and patient tumors generated tumor spheroids directly from patient samples that were first mechanically and enzymatically disaggregated then filtered to be between 40 and 100 μm in size before being suspended in a collagen scaffold and maintained in a microfluidic chamber. These spheroids represent small *ex vivo* tissue explants containing a mixture of cells from the original biopsy, including the endogenous immune cell complement. In this case, these models were used to examine immune checkpoint blockade, identifying that TBK1/IKKε inhibition enhanced response to programmed cell death protein 1 blockade and matched tumor response *in vivo* (Jenkins et al., 2018). The same group adapted this apparatus utilizing spheroids generated from established cancer cell lines in this collagen scaffold microfluidic device and introduced T cells (Jurkat) into the media (Kitajima et al., 2019). The infiltration of T cells into the spheroids was studied and identified that tumor spheroids lacking liver kinase B1, which represent a subset of tumors that are resistant to immune checkpoint blockade, impaired the recruitment of CXCR3-expressing Jurkat T cells (Kitajima et al., 2019).

Organoid co-culture models of the TME have also been used to explore immunotherapy approaches *via* direct tumor organoid-T-cell co-culture systems and also investigate the phenomenon of immune evasion (Sharma et al., 2017; Dijkstra et al., 2018; Cattaneo et al., 2020). A model examining tumor infiltrating lymphocytes in biopsy-derived rectal tumor organoids supplemented with patient-matched lymphocytes was predictive of patient response to neoadjuvant chemoradiotherapy (Matsutani et al., 2018). Moreover, patient-derived tumor organoids co-cultured with patient-matched CAFs and T-cells also demonstrated enhanced chemoresistance to gemcitabine compared to tumor organoids in monoculture (Tsai et al., 2018). These findings, amongst others, highlight CAFs as promising potential therapeutic targets to treat PDAC.

An additional use of the co-culture platform with respect to the immune system is the examination of chronic inflammation. Chronic inflammation accounts for 15%-20% of cancer deaths, and typically can manifest in the forms of inflammatory bowel disease in colorectal cancer, or in hepatocellular carcinoma *via* hepatitis B or C infection (Coussens and Werb, 2002; Mantovani et al., 2008). There is reciprocal cross-talk between precursors to cancer cells and inflammatory cells and therefore represents a potential area of study that co-culture studies may help to explore tumor initiation (Mantovani et al., 2008; Diakos et al., 2014). Existing infection models such as *H. pylori* infection of gastric organoids identified that gastric organoid production of the inflammatory cytokine IL-8 along with urease acts as a chemoattractant for *Helicobacter pylori* which then delivers the transforming agent CagA to the epithelium (Bartfeld et al., 2015; Huang et al., 2015). Further, *Escherichia coli* exposure to intestinal organoids can induce oncogenic mutations (Pleguezuelos-Manzano et al., 2020).

These examples demonstrate the versatility of co-culture systems to examine the interplay between cancer cells and various aspects of the immune system, including the role of inflammation in tumor initiation. Co-culture platforms permit effects of specific immune cell types to be studied in isolation, which is difficult to do *in vivo*.

### 3.3 Vasculature and angiogenesis

Another clinically important aspect of tumor development is novel angiogenesis to support the tumor growth. Indeed, a major class of cell types present in the TME are tumor endothelial cells, which contribute to tumor progression by means of neovascularization and provide routes for blood-borne metastasis (Senger et al., 1983; Folkman, 1985; Folkman et al., 1989; Armulik et al., 2005; Hanahan and Coussens, 2012; Ayuso et al., 2016; Hida et al., 2018). Vasculature in cancer tends to be poorly organized, which can be due to pericyte depletion, and porous compared to that in healthy tissue, allowing perfusion of nutrients whilst gaps facilitate tumor cell migration out from the primary tumor site (Cooke et al., 2012; Dudley, 2012; Thomas et al., 2017; Hida et al., 2018).

In concert, tumor cells and CAFs secrete pro-angiogenic factors such as VEGF, FGF2, PDGFA and PDGFB (Eiro et al., 2018). Newly synthesized blood vessels are required to sustain high grade tumors as their cores become hypoxic and nutrient deficient. There are several models that examine angiogenesis by co-culture of epithelial cancer cells together with endothelial cells, including microfluidic and micro-tissue constructs (Chaddad et al., 2017; Mazio et al., 2018; Pradhan et al., 2018; Shirure et al., 2018; Nashimoto et al., 2020). *In vitro* models of angiogenesis in the non-tumor context also exist for comparison (Fuchs et al., 2007; Bischel et al., 2013).

In the breast cancer context, a multi-channel microfluidic co-culture arrangement of fibroblasts and endothelial cells in a central chamber with tumor cells in adjoining chambers was used to examine angiogenic sprouting (Shirure et al., 2018). CAFs, cancer cell lines and patient derived tumor organoids, but not normal fibroblasts, heavily induced angiogenesis, and the platform could also be used to assess chemotherapeutic efficacy both against the cancer cells (paclitaxel), but also the effect of anti-angiogenic compounds (bevacizumab) (Shirure et al., 2018). Another multi-channel microfluidic arrangement determined that colorectal cancer spheroids upregulated the expression of angiogenic genes when in co-culture with fibroblasts (Jeong et al., 2016).

Studies have identified that cancer cells on their own may not be sufficient to induce angiogenesis. A model for inducing angiogenesis from HUVEC cells in side-channels in a microfluidic device identified that neither breast cancer spheroids nor colorectal cancer spheroids were capable of inducing angiogenesis in monoculture (Nashimoto et al., 2020). Angiogenesis in this model with cancer spheroids required co-culture with lung fibroblasts, which themselves incidentally could mildly induce angiogenesis in monoculture (Nashimoto et al., 2020). Curiously, when HUVECs were themselves also incorporated into the spheroids, as a tri-culture of cancer cells, fibroblasts and HUVECs, the angiogenic potential of the spheroids were reduced compared to the co-cultures of only cancer cells and fibroblasts (Nashimoto et al., 2020). In addition, co-culture assays have been developed for quantification of angiogenesis that can be adapted to screen for novel anti-angiogenic compounds or even used in a personalized medicine context by screening known anti-angiogenic therapeutics against patient-derived tumor organoids (Li S. et al., 2018; Truelsen et al., 2021). Altogether, these co-culture models are a useful tool for studying angiogenesis as well as identifying effective therapeutics and underscoring the importance of multi-cellular interactions in this process.

### 3.4 Metastasis

Metastasis, whereby cancer cells leave the primary tumor site and establish at secondary sites in the body is a clinically critical step in tumor progression. The process of metastasis is multi-step and involves epithelial to mesenchymal transition (EMT), an increase in invasive characteristics leading to intravasation into the blood stream or lymphatic systems and finally the successful colonization at a suitably receptive site (Alizadeh et al., 2014). Typically, a tiny proportion (0.001%-0.02%) of cells that undergo intravasation are successful at establishing a metastatic tumor, however successful metastasis is a severe risk factor that worsens prognosis considerably (Weilbaecher et al., 2011).

There are several examples of cancer cell-fibroblast co-cultures that demonstrate an increase in cancer cell invasive characteristics by direct observation of cancer cell migration. One example using lung cancer cells co-cultured with either normal or cancer associated fibroblasts identified that tumor cell proliferation was increased by both types of fibroblasts compare to cancer cells in monoculture. However, only when co-cultured with CAFs did the cancer cells begin to invade into the collagen gel they were cultured on (Horie et al., 2012). A microfluidic model of breast cancer exploring the transition of ductal carcinoma *in situ* (DCIS) to invasive ductal carcinoma identified that cancer cells appeared to undergo a biphasic transition to the invasive phenotype, where cancer cells in co-culture underwent a morphological change associated with invasion, but only those cancer cells that were very close to the fibroblasts became fully invasive. This was also associated with matrix remodeling (Sung et al., 2011). Another 3D microfluidic model of breast cancer transition of DCIS to invasive ductal adenocarcinoma

Observed a similar phenomenon whereby cancer cell co-culture with fibroblasts was required to induce an invasive phenotype of cancer cells, whereas control, non-cancer, epithelial cells never showed signs of invasion regardless of co-culture (Bischel et al., 2015). The induction of an invasive phenotype is not just a result of interactions with CAFs, however. For example, indirect transwell co-culture of breast cancer cells with adipocytes resulted in increased invasion of cancer cells and facilitated by an associated change in energy metabolism favoring fatty acid oxidation which, when blocked, inhibited cancer cell invasion (Wang et al., 2017).

Interactions with the tumor-stroma also contribute to EMT, motility and invasion in the case of non-small cell lung cancer (Choe et al., 2013). In this example, an established lung cancer cell line was co-cultured with CAFs isolated from lung cancer biopsies in direct and indirect 2D conditions. Cancer cells indirectly co-cultured with CAFs proliferated more than when co-cultured directly, however direct co-cultures more potently induced EMT markers and cell motility (Choe et al., 2013).

Another aspect to metastasis is the suitability of site reception of the metastasized cancer cells. One model of lung colonization by cells of different cancer types (ovarian and colon) used a co-culture spheroid model of lung architecture by mixing together normal lung epithelial cells together with lung fibroblasts, HUVECs and human normal lymphatic endothelial cells in ultra-low attachment plates called “primitive lung in a dish” (PLiD) (Ramamoorthy et al., 2019). Cancer cell lines were directly incorporated into PLiDs to model metastasis to the lung. Introduced cancer cells colonized PLiDs and rates of colonization were enhanced when lung spheroids were pre-treated with tumor exosomes. Chemotherapeutic responses of patient derived cancer cell lines in PLiD metastasis models were also assessed where responses matched those seen in patients.

Overall, these examples demonstrate that there are a variety of co-culture models that are useful to study the process of metastasis of cancer cells both in the primary tumor and also for their establishment at secondary, distant sites.

## 4 Designing a co-culture; guidelines and reporting

As shown above, co-cultures are powerful tools to study a multitude of aspects of cancer biology. Manipulation of many variables can give rise to complex model systems. We have identified several key factors important to consider when constructing a co-culture experiment, and suggest that clear reporting of these factors is crucial in promoting robust, reproducible experimental data that can be readily contextualized in the surrounding literature (Table 2).

**TABLE 2:** Guidelines for co-culture design and features to include when reporting; a check-list. A summary of the three key aspects identified for co-culture model construction and a fourth key aspect describing model evaluation for validation. Each key aspect is itemized with pertinent considerations for co-culture model design as well as a designation on features that need reported to contextualize co-culture findings.

Key aspects	Considerations	Report
i) Cell Types and interactions	Cancer cell	Species and tissue background
		Confirm cell-specific characteristics
		Confirm cell culture purity (no contamination)
	Stromal cell type(s)	Species (if different, xeno-co-culture), tissue background and cell type
		Confirm cell-specific characteristics
		Confirm cell culture purity (no contamination)
	Juxtacrine/Paracrine Interactions	Direct or indirect co-culture
	How to identify cell types later, if required?	Any cell modifications performed, markers used
ii) Physical arrangement and ECM context for each cell type	Dimensionality	Whether 2D, 3D or a combination
	Physical cell context; Mechanotransduction	Description of co-culture architecture. Culture plastic, membrane, spheroid/tumor organoid, Scaffold (free) details
	ECM biochemical context	Description of chemical background of the co-culture; Inert or active?
iii) Media	Does the media support the cells?	Effect of co-culture media selection on mono-cultured cell types included
	Does the media influence relevant signaling?	Appropriate control conditions; consider different media for comparison—is effect dependent on particular medium?
	Media exchange (Microfluidics)	Static or dynamic media regimen; description
		Physical characteristics (Such as pressure, flow rate)
		Body-on-a-chip; serial chambers and order, potential conditioned media effects of preceding chamber
iv) Validation	How well does the model perform?	How do adapted models compare to existing *in vitro* models?
		For models with no existing *in vitro* comparison; assess by comparison to *in vivo* situation
	Predictive capacity and translation	Do model results make predictions that can be assessed *in vivo*? How do these compare?

A useful co-culture ought to be designed to examine physiologically relevant interactions, which can be achieved by considering several design principles outlined here. Foremost to consider is the cell types required to model the biological question of interest, including examination of whether paracrine, juxtacrine or a comparison between the two are required. It is critical that each cell type is validated properly in advance of co-culture to support interpretation and reproducibility of results. Proper cell validation is to ensure that *in vitro* cell types still share characteristics with their *in vivo* counterparts. Whilst absolute proof of identity is typically impossible, cells still ought to demonstrate characteristic, phenotypic behaviours, and retain cell-specific markers and responses to key stimuli which can be assessed by morphological or transcriptomic means, for example, transcriptional analysis of marker genes and their fluctuation in response to described stimuli. In cases where artificially immortalized cell lines are used, a check that they have not become fully transformed is prudent and can be assessed by transformation assays (Creton et al., 2012; Mascolo et al., 2018). Consideration should also be paid to cell population purity and other sources of heterogeneity that may influence cell behaviours prior to their incorporation to a co-culture, which may contribute additional complications to understanding experimental results. It is also critical to consider the species used in a co-culture model and especially if a xeno-co-culture is designed, combining cell types from multiple species. These factors are critical for interpreting experiments and introduces an additional barrier to validation for translational efforts. Further, downstream applications may require identification of specific cell types; appropriate methods may include antibody staining or modification of cell lines to express different fluorophores as examples. If modifications to the cells are performed a consideration of whether this modification may alter cellular behaviour is required such as is a risk with lenti-viral introduction of fluorophore sequences *via* random integration into the genome as an example.

**FIGURE 4:**
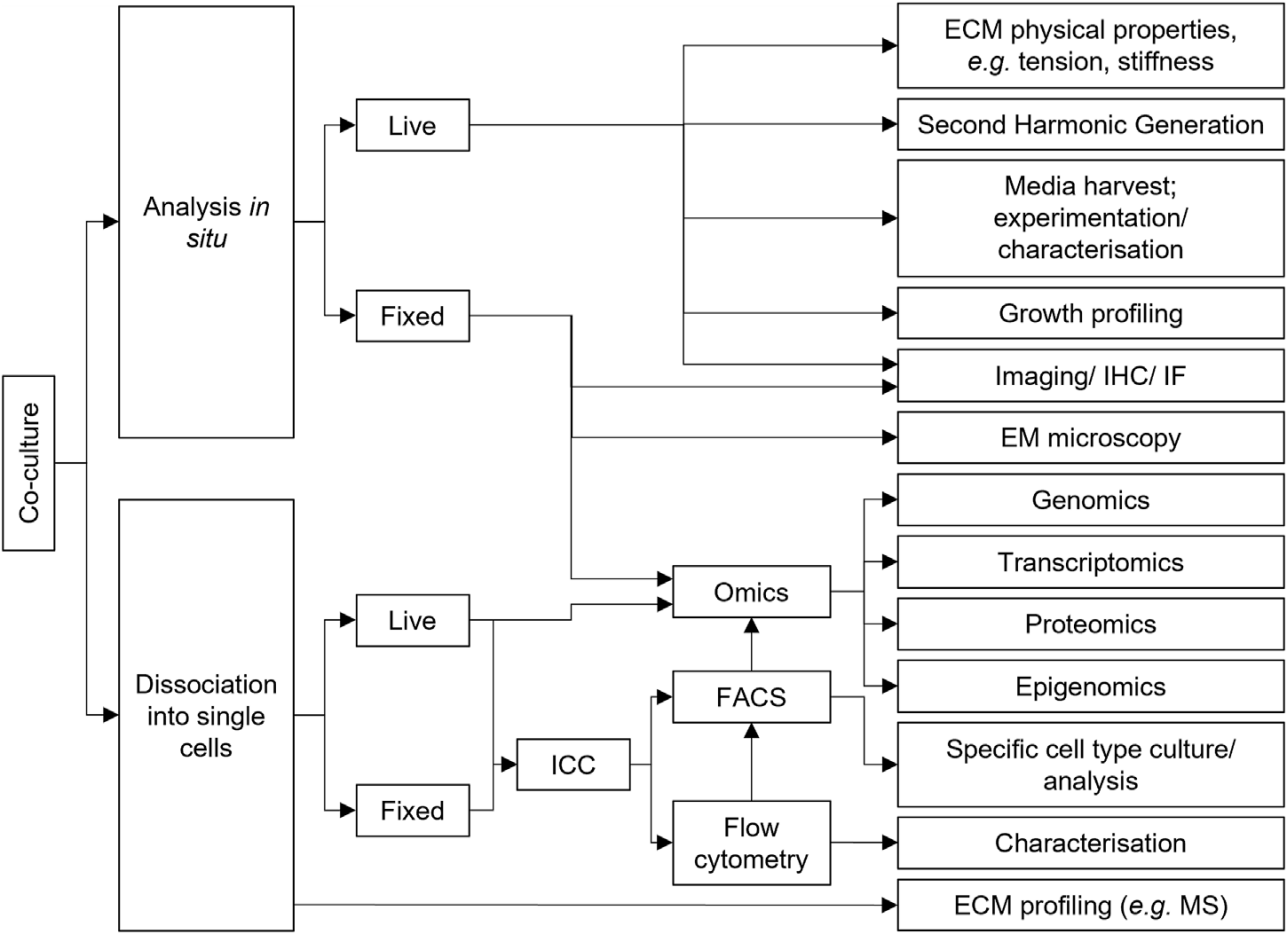
Co-culture experimental versatility. A non-exhaustive selection of assays that can be performed with co-culture models. These assays can be used to determine what the effect of co-culturing cell types together is compared to mono-cultured counterparts to identify biologically relevant interactions and mechanisms. Arrows represent typical workflows. ECM, Extracellular matrix; EM, Electron microscopy; FACS, Fluorescence activated cell sorting; ICC, Immunocytochemistry; IF, Immunofluorescence; IHC, Immunohistochemistry; MS, Mass spectrometry.

Once the desired biological question and interaction of interest is determined, it is important to consider what type of co-culture best addresses the hypotheses, especially with the analytical readout approach in mind. As indicated by the preceding sections, co-cultures can vary immensely by design, however ultimately, as with any assay, the ability to measure variables pertinent to addressing hypotheses is critical to any model system. Indeed, co-culture models are experimentally versatile and readily amenable to a plethora of assays for investigating cell behaviour (Figure 4). Co-cultures can be analysed by existing assays used in mono-culture models, such as imaging, immunohistochemistry (IHC), immunofluorescence (IF) immunocytochemistry (ICC), cell sorting, and the gamut of omics techniques, amongst others, in order to determine biological insight.

However, despite their versatility, co-cultures also demonstrate unique challenges compare to mono-culture assays. For instance, destructive assays on bulk cultures, including protein (such as for ELISA or western blots) or RNA extraction (such as for PCR or RNAseq) from co-cultured, will lose information on which cell type produced any change in protein or RNA transcript of interest. This limitation can be partially address by cell sorting approaches or even single cell analysis techniques to robustly identify the cell type in which biological change is pertinent. However, even then, spatial relationships can be lost in these cases, where co-cultured cells may behave differently based upon their proximity to the other cell types in the culture. As a result, analyzing co-culture systems, which represent more complicated model systems that contain multiple feedback loops between cell types, becomes a more complicated endeavor. Furthermore, different co-culture approaches have different challenges. For example, bioprinted and microfluidic/organ-on-a-chip systems typically use extremely small volumes of material and media compared to, for instance, scalable bioreactor-based assays. Consequently, material availability can be limiting and may influence the analytical method adopted. Ultimately, care in experimental co-culture design requires forethought for compatibility with the analytical tools envisaged for addressing purported hypotheses.

Next is consideration of the physical framework of the model such as the dimensionality of the co-culture. It is technically easier and considerably less expensive to use 2D arrangements. Part of the ease of a 2D arrangement is that many cell culture analysis tools are designed with traditional 2D cell culture in mind, although there has been considerable improvement in the development of tools designed for examination of 3D cell cultures. That said, the advantages of 3D cultures are readily identifiable; permitting the self-determined arrangement of cells whether in mono- or co-culture that are less prone to genomic instability. In addition, the ECM context is crucial with respect to mechanotransduction and chemical signaling. However, there are some assays that may be sensitive to the ECM context employed. For example, proteomic analyses can become challenging in assays where cells are cultured in scaffolds with a high protein content that consequently reduce the signal compared to the background provided by that scaffold. Alternatively, scaffold selection may impact on readouts due to contribution of autofluorescence. Consequently, it is crucial to ensure that scaffold choice is compatible with experimental read-out and whatever ECM context is used, it must be clearly communicated.

Choice of media is another factor that is crucial in co-culture design. As outlined previously, the best selection for every context is often not clear and complications may arise where analogous cell types require different media formulations between species. However there are some considerations that can help guide selection. Firstly, whether a candidate medium can sustain all the cell types in a co-culture; which can be tested by mono-culture of all cell lines in candidate media. Of course, such as in the case of symbiotic rescue experiments, that media alone is insufficient to maintain a particular cell line may be the observation under investigation. However, for many monoculture cell lines, and especially for difficult-to-culture cell lines, media is tailored towards the sustained propagation of that particular cell type. Introducing different cell types into these conditions may not be appropriate for examining their behaviour. Indeed, a particular confounder is when media is tailored towards supporting the proliferation of cells that are normally non-proliferative *in vivo*, potentially adding a confounder to interpreting cell behaviours. To add to the complexity of media selection are the microfluidics technologies, which introduce the capacity to model other physiologically relevant factors such as pressure and continuous flow that permit the development of dynamic co-culture models.

Finally, once a model is constructed, arguably the most difficult aspect is model validation. There are now a great many models out there for examining co-culture behaviours, and whilst the authors strongly recommend building knowledge using existing tools, they recognize that this is not always feasible. That said, for new models, features that are comparable with existing models could be examined to check for idiosyncratic discrepancies in model behaviour that could influence biological interpretation. Alternatively, comparison or validation of the model with the *in vivo* setting is also a powerful way to validate a model. However, the gold standard for effective *in vitro* modelling is whether predictions generated by the model are verified *in vivo*.

## 5 Take home messages

Tumor-stroma interactions *in vivo* are complex. Necessarily the use of models is essential to interrogate the biology, and to identify targets for drug development. Work on biopsy materials is cumbersome, slow, and not easily controlled. To overcome these limitations, animal models have been developed that have greatly contributed to our understanding, but they are expensive, time consuming and may not be directly translatable; “*mice are not small people*” (Rangarajan and Weinberg, 2003). Aside from patients, animal models are the most complex and holistic way of analyzing medically relevant problems and techniques such as patient derived orthotopic xenografts capitalize on the advantages of clinically relevant patient material together with systemic and/or interventionist studies in animal models that can identify patient specific treatment strategies (Hidalgo et al., 2014; Fernández-Rodríguez et al., 2020; Zhang et al., 2020; Abdolahi et al., 2022). At the opposite end of the scale, cell line monoculture is the most reductionist way of analyzing cell biology. For the cell lines established, monoculture is both cheap to run and extremely scalable however, these too, lack as effective models, since more than 90% of therapeutics discovered in 2D cell lines are not clinically viable (Adams, 2012; Hay et al., 2014; Imamura et al., 2015). This is for a number of reasons already discussed. Contributing factors for this lack of translatability include effective modelling of the microenvironment, including appropriate ECM composition, mechanical factors, neighboring cell interactions, inter-organ and systemic factors. In between the extremes of holistic and reductionist approaches fit organoid, co-culture and microfluidic techniques. Here, the aforementioned TME features can be incorporated to permit both scalable and reproducible assays as well as potential for providing real-time guidance in treatment strategies using patient derived tumor organoids (Verissimo et al., 2016; Yang et al., 2018). It is clear that many different groups are working to incorporate these different variables, with the intention of generating better models.

Technological innovation has facilitated the manifestation of much creative cell-culture modelling. Now there are many models of the tumor-stroma and more are being developed all the time. While the creativity of the models generated is to be commended, there is a risk that the field leans heavily towards *de novo* generation rather than utilizing tools and models that already exist. This can impede reproducibility and comparison between research groups. This is of particular concern for models that utilize custom 3D printed structures in, for example, microfluidic models or other synthetic scaffolds. The authors believe building on research with existing tools is likely to drive the field forward more quickly and that that new models are best developed when existing tools are insufficient to address specific biological questions. Further, where new models are presented, groups should also validate their findings on existing models to support consistency and reproducibility of findings.

Whichever model is selected; research groups must make it clear what conditions are being tested. Clarity when reporting the key aspects, or building blocks highlighted in this review, will contribute to robustness and help to contextualize research between groups. Critically, papers often publish models with vague or incomplete media descriptions which means that the work *cannot* be externally validated.

An overarching theme across all these epithelial, stroma-rich cancers are the multifaceted contributions of different cell types. Take, for example, the consideration of the many types of CAFs that differentially influence their respective tumors. Identifying the key functions of each CAF subtype cannot readily be done outside of *in vitro* models. Elucidating these specific interactions and intelligently manipulating the stromal composition of these tumors could be a key to therapy, and is a phenomenon readily amenable to investigation and validation in such co-culture models. Furthermore, these model systems are ideally suited for high throughput screening assays for novel therapeutic identification by disrupting the tumor promoting aspects of the relationship between cancer cells and stroma in the TME. This is an area where these fascinating technologies can excel.

Existing models are valuable assets that can be utilized by researchers in this complex field, and there is vast opportunity for researchers to push for answers to the outstanding biological questions at the juxtacrine, paracrine, endocrine and systemic levels: what can we learn from co-cultures, how do we untangle their complex web interactions, and how, ultimately, can we use this knowledge for the benefit of patients?

## Glossary

α-SMA: alpha-Smooth muscle actin
2D: Two dimensional
3D: Three dimensional
ABCG2: Adenosine triphosphate-binding cassette super-family G member 2
CAF: Cancer Associated Fibroblast
CD4: Cluster of differentiation 4
CXCR3: C-X-C motif chemokine receptor 3
DCIS: Ductal carcinoma *in situ*
ECM: Extra-cellular matrix
ELISA: Enzyme-linked immune sorbent assay
EMT: Epithelial-mesenchymal transition
FGF: Fibroblast growth factor
GRP78: Glucose regulated protein 78
HSF1: Heat-shock transcription factor 1
HUVEC: Human umbilical vein endothelial cells
iCAF: Inflammatory cancer associated fibroblast
ICC: Immunocytochemistry
IF: Immunofluorescence
IFN: Interferon
IHC: Immunohistochemistry
IKKε: Inhibitor of nuclear factor kappa-B subunit epsilon
IL: Interleukin
JAK: Janus Kinase
LIF: Leukaemia inhibitory factor
LIN28: Lin-28 homolog A
MCF7: Michigan cancer foundation-7
MMP: Matrix metalloproteinase
myCAF: Myofibroblastic-like cancer associated fibroblast
PCR: Polymerase chain reaction
PDAC: Pancreatic ductal adenocarcinoma
PDE: Patient derived explant
PDGF: Platelet-derived growth factor
PD-L1: Programmed death ligand 1
PLiD: Primitive lung in a dish
RNA: Ribonucleic acid
RNAseq: Ribonucleic acid sequencing
SOX2: Sex determining region Y-box 2
STAT: Signal transducer and activation of transcription
TBK1: Tumor necrosis factor receptor associated factor family member-associated nuclear-factor-kappa-B activator-binding kinase
TGFβ: Transforming growth factor beta
TIMP: Tissue inhibitor of matrix metalloproteinases
TME: Tumor microenvironment
TNBC: Triple negative breast cancer
VEGF: Vascular endothelial growth factor
